# Secondhand tobacco smoke exposure in homes and vehicles in youth: disparities among racial, and sexual and gender minorities

**DOI:** 10.3389/fpubh.2024.1370552

**Published:** 2024-07-23

**Authors:** Rajesh Talluri, Sahil S. Shete, Surendra S. Shastri, Sanjay Shete

**Affiliations:** ^1^Department of Biostatistics, The University of Texas MD Anderson Cancer Center, Houston, TX, United States; ^2^Department of Behavioral Science, The University of Texas MD Anderson Cancer Center, Houston, TX, United States; ^3^Department of Health Disparities Research, The University of Texas MD Anderson Cancer Center, Houston, TX, United States; ^4^Department of Epidemiology, The University of Texas MD Anderson Cancer Center, Houston, TX, United States; ^5^Division of Cancer Prevention and Population Science, The University of Texas MD Anderson Cancer Center, Houston, TX, United States

**Keywords:** secondhand tobacco smoke exposure, racial disparities, sexual minorities, adolescents, National Youth Tobacco Survey

## Abstract

**Background:**

Secondhand smoke exposure (SHSe) among youth is a serious public health concern, leading to an increased risk of conditions such as asthma and respiratory infections. However, there is little research on SHSe among vulnerable populations, such as racial and sexual minorities. Understanding the factors associated with youth SHSe in homes and vehicles is crucial to developing better protective policies.

**Methods:**

This study utilized 2020 data from the National Youth Tobacco Survey, a representative sample of middle- and high-school students in the US. The primary outcomes were youth SHSe at home and while riding in a vehicle. Multinomial regression models were used to assess factors associated with SHSe.

**Results:**

The data included 9,912 students enrolled in grades 6 through 12 in the United States who reported never using any form of tobacco. Non-Hispanic Black students living with someone who does not use any form of tobacco products were significantly more likely to experience moderate [OR = 2.1 (1.1–3.9), *p* = 0.03] and severe [OR = 5.1 (2.2–11.7), *p* < 0.001] secondhand smoke exposure (SHSe) in homes compared to their non-Hispanic White counterparts. Heterosexual female students had lower odds of reporting moderate SHSe in the home compared to heterosexual males [OR = 0.7 (0.6–0.99), *p* = 0.02], whereas bisexual females had two-fold increased odds of severe SHSe in homes [OR = 2.0 (1.2–3.4), *p* = 0.01].

**Conclusion:**

Significant efforts are needed to develop targeted interventions to reduce SHSe in homes and vehicles, particularly in these vulnerable populations.

## Introduction

Secondhand smoke exposure (SHSe) causes serious health issues in non-smoking adults and children as they are inhaling many of the same harmful toxins as active smokers do (1–5). Longer durations and higher levels of SHSe can increase the risk of lung cancer (3, 4). SHSe has been reported to lead to several conditions in children, such as more frequent and severe asthma attacks, respiratory infections, impaired lung functions, and ear infections (3, 4, 6). SHSe has also been associated with a higher risk of ischemic heart disease, stroke, and type 2 diabetes (4). A systematic literature review found that prenatal or postnatal SHSe was associated with a risk of lower birth weight, stunted height, wasting, and a lower head circumference (7). Children who lived with a smoker for over a decade were associated with having higher mortality from chronic obstructive pulmonary disease (8).

There are several laws at local and state levels to ensure that all non-hospitality workplaces, restaurants, and bars are 100% smoke-free that have been successful in reducing SHSe (9). Public smoking bans have been beneficial in decreasing incidents of acute coronary events such as heart attacks and acute myocardial infarctions (10). Furthermore, The U.S. Surgeon General recommends that parents protect their families by not allowing smoking anywhere in their homes or cars, ensuring their children’s schools are tobacco-free, and avoiding locations that allow smoking (2). SHSe in areas such as homes and vehicles is especially dangerous, as tobacco smoke in enclosed spaces can produce extremely unhealthy levels of Particulate Matter 2.5, an air pollutant that can negatively impact respiratory and cardiovascular function (11).

Adolescent never-smokers exposed to secondhand smoke at home are also at an increased risk for initiating smoking compared to those not exposed (12). Many states have implemented smoke-free policies, particularly in subsidized and public housing, to minimize health risks. However, enforcement remains inconsistent, and some states rely on voluntary compliance by landlords, limiting the impact of these policies. As of December 31, 2023, only 16 states have enacted smoking restrictions for public or private multi-unit housing. Among these, 14 of the states restrict smoking in common areas only, despite the risk of secondhand smoke infiltrating residential units from other spaces (13).

Evidence also suggests that SHSe in a motor vehicle may lead to nicotine-dependent symptoms (e.g., physical and mental cravings, susceptibility to environmental cues) in 10–12 year-olds (14).

Importantly, there is broad support for prohibitions on smoking in vehicles when children under the age of 13 are present (15). However, only thirteen states specifically prohibit smoking in vehicles used to transport children in childcare facilities. Only, 11 states prohibit smoking in personal vehicles when children are present (16).

The US surgeon general’s recommendation and existing state laws aimed at reducing SHSe have been effective in reducing SHSe prevalence in the US (87.5% in 1988 to 25.3% in 2012) (17), however, they have stagnated in following years (25.3% in 2012 to 24.6% in 2018), and inequalities still exist in particular demographics (4, 17–19). According to 2011–2018 National Health and Nutrition Examination (NHANES) data (18), SHSe was higher among youth aged 3–11 and 12–19 compared to adults over 20 years old. Non-Hispanic Black individuals also had a higher prevalence of exposure compared to non-Hispanic White individuals and Mexican Americans. Furthermore, those living below the poverty level had over two-fold increased prevalence compared to those who live at or above it. SHSe prevalence for renters was also double compared to those who owned their homes. Lastly, those who lived with a smoker in the home also had a higher prevalence compared to those who lived in a home with no smokers (18).

The prevalence of tobacco use varies by sexual orientation identity (20, 21). The use is substantially higher among sexual and gender minorities compared to heterosexual individuals (22). Furthermore, individuals who identify themselves as bisexual have a higher cumulative incidence of starting smoking at an earlier age compared to heterosexuals (20). The high tobacco use is attributed to be a coping mechanism brought upon by the stress and stigma (23). Although there have been several studies assessing tobacco use and trends among sexual minorities, little is known about SHSe in this vulnerable group.

SHSe also creates a burden on the healthcare system and economy. Research shows that healthcare costs from SHSe are declining. However, the costs are still substantial and avoidable ($4.6 billion in 2000, $2.1 billion in 2005, and $1.9 billion in 2010) (24). Overall, SHSe resulted in an estimated 42,000 deaths and $6.6 billion of lost productivity in 2006 (25) and $6.5 billion loss in 2009, which is equivalent to $8.2 billion in 2017 dollars (26). School children with an adult(s) who smoked in the home were more likely to have school absences than those who did not live with smokers, which is valued at an estimated $227 million loss in their caregivers’ work productivity (27).

There is strong public support for implementing smoke-free policies to keep children safe, with the highest levels of support found for places frequented by children, such as cars carrying children (86%) and playgrounds (80%) (28), particularly, with non-smokers, former smokers, and women showing higher levels of support. Despite official recommendations, successful public policies, and individual support, SHSe in private spaces remains a concern. A report utilizing the 2016 National Youth Tobacco Survey found that 29% of U.S. youth were exposed to SHSe at least one day during the past 7 days at home or in a vehicle SHSe (29). Another study (30) utilizing the 2019 National Youth Tobacco Survey data reported that SHSe prevalence at homes was 25.3% and in vehicles was 23.3% among US middle and high school students. The report also found that SHSe in homes declined significantly from 2011–2018, except for non-Hispanic Black students. Even though these studies evaluated the prevalence of SHSe, the degree and severity of SHSe and the associated factors in different subpopulations have not been studied in the literature. We hypothesize that the degree of SHSe will significantly vary among racial, sexual, and gender minorities.

Overall, children experience SHSe more frequently than adults, and it most frequently occurs within the home (31). Even when young individuals abstain from tobacco products, they can still be exposed to SHSe in situations beyond their control, such as in family homes and vehicles. Therefore, this study aims to identify racial, sexual, and gender disparities in exposure to secondhand smoke among youth in homes and while they ride a vehicle. Addressing these disparities is vital for developing effective public health interventions and protecting vulnerable populations.

## Methods

### Data and sampling design

This study utilized data from the 2020 cycle of the National Youth Tobacco Survey (NYTS). NYTS is a cross-sectional survey developed to collect data to evaluate tobacco prevention and control programs and is representative of middle and high school students in the United States (32). The survey design of NYTS consists of a three-stage cluster sample design. The first stage samples primary sampling units, which are counties or a group of small counties; the second stage comprises selecting secondary sampling units, which are schools within each of the selected primary sampling units; and the third stage comprises selecting classes within each grade level of the selected school. The survey was administered to all students within a selected class. Participation in the NYTS is voluntary at both the student and school levels. The survey design was stratified by several factors at each sampling stage. The primary sampling units were stratified by race/ethnicity and urban vs. non-urban designation. Then, the schools were stratified by their size (small, medium, and large) and educational level (middle school vs. high school). The survey data was collected electronically, maintaining confidentiality. The 2020 NYTS survey data was rigorously checked to confirm its representativeness and minimize bias despite data collection being interrupted due to COVID-19. The sample was verified against various demographic, geographic, and socioeconomic characteristics to ensure precise estimates for key subgroups. Specifically, the sample was confirmed as representative by comparing the distribution of participating schools with the broader subset of agreeing schools across U.S. regions (South, East, Midwest, and West), school types (public and non-public), and educational levels (middle and high schools).

This study followed the Strengthening the Reporting of Observational Studies in Epidemiology (STROBE) reporting guideline. The Office of Management and Budget (OMB), ICF’s Institutional Review Board (IRB), and CDC’s Institutional Review Board (IRB) approved the NYTS cycles used in this study. Because the NYTS data were deidentified and publicly available, our secondary data analysis was exempt from institutional review board approval.

### Outcome

The primary outcomes of this study were youth exposure to secondhand tobacco smoke at home and while riding in a vehicle. The study population included only those students who have never used any form of combustible or noncombustible tobacco products in order to avoid confounding responses from smokers and to characterize the smoke exposure as purely secondhand. The primary outcomes were obtained from two questions: “During the past 7 days, on how many days did someone smoke tobacco products in your home while you were there?” and “During the past 7 days, on how many days did you ride in a vehicle when someone was smoking a tobacco product?.” The responses for both questions were categorized into three levels: (1) No Exposure: Exposed for 0 days in the past 7 days; (2) Moderate Exposure: Exposed for 1–4 days in the past 7 days; (3) Severe Exposure: Exposed for 5–7 days in the past 7 days.

### Statistical analysis

Because the NYTS is based on a three-stage cluster sampling design, survey-adjusted weights were used to estimate the prevalence of SHSe. The base sampling weight for each student was calculated using the inverse probability of selection at each stage. These base weights were adjusted for nonresponse by sex and grade level within each school. These weights were then further adjusted using a poststratification approach to match national estimates of student counts in middle and high schools by age, sex, and race/ethnicity categories.

As the two outcomes of interest had three levels, we employed a survey-weighted multinomial regression approach to identify factors associated with SHSe in homes and while riding a vehicle. Multinomial regression was chosen as the appropriate statistical method because the outcome variables are categorical with more than two levels, and the model allows for the simultaneous comparison of multiple outcome categories without assuming the proportionality of the odds ratios. All variables included in the models were selected *a priori* based on their significance and relevance to the research question. It is important to note that we did not conduct stepwise or any other model selection processes, as these approaches can inflate the type 1 error rate (33). The ‘svymultinom’ method from the R package ‘svrepmisc’ was used to model the multinomial regression. These analyses were adjusted for age, sexual/gender identity, educational level, race/ethnicity, and whether or not the students were living with someone who used tobacco products. All analyses were performed using the ‘survey’ package in R version 4.0.3. The significance level was calculated using a two-sided Wald test for all statistical analyses and defined as *p* ≤0.05.

## Results

### Characteristics of the study population

The data from the 2020 NYTS included 14,531 students (Weighted *N* = 27,563,807) enrolled in grades 6 to 12 in the United States. Among these, 9,912 students (Weighted *N* = 18,447,190) reported they never used any form of tobacco and, thus, were the study population of interest. Table 1 shows the composition of the study population by select characteristics. 47.5% were non-Hispanic White individuals, 12.5% were non-Hispanic Black individuals, and 25.8% were Hispanics. 49.8% were female, 4.3% self-identified as bisexual females, and 39.7% self-identified as heterosexual females. Among those who did not live with a tobacco user in their homes, 32.8% were non-Hispanic White individuals, 8.2% were non-Hispanic Black individuals, and 19.9% were Hispanics. Whereas among those who lived with a combustible tobacco user in their homes, 12.8% were non-Hispanic White individuals, 3.2% were non-Hispanic Black individuals, and 5.4% were Hispanics.

**Table 1:** Survey weighted prevalence of select characteristics among middle- and high-school students who have never used any form of tobacco—National Youth Tobacco Survey, 2020.

Variable	Percentage (95% CI)	*N* (Weighted *N*)
SSE at home		
No exposure	84.9 (83.2–86.4)	8,255 (15437260)
Moderate exposure	7.5 (6.5–8.5)	738 (1358980)
Severe exposure	7.7 (6.8–8.6)	766 (1394543)
SSE in car		
No exposure	87.6 (86.1–89.0)	8,477 (15854114)
Moderate exposure	8.7 (7.7–9.7)	850 (1567378)
Severe exposure	3.7 (3.1–4.4)	375 (671546)
Sex		
Female	49.8 (48.4–51.2)	5,033 (9161147)
Male	50.2 (48.8–51.6)	4,852 (9233994)
Sexual identity		
Heterosexual	82.9 (81.6–84.0)	7,936 (14876371)
Gay or lesbian	2.2 (1.9–2.6)	207 (400768)
Bisexual	5.5 (4.8–6.3)	519 (992727)
Not Sure	9.4 (8.2–10.6)	942 (1679606)
Sex-sexual identity		
Male-heterosexual	43.3 (41.8–44.8)	4,044 (7755252)
Female-bisexual	4.3 (3.7–4.9)	399 (765330)
Female-gay or lesbian	1.3 (1.0–1.6)	121 (227684)
Female-heterosexual	39.7 (38.1–41.3)	3,886 (7112231)
Female-not sure	4.7 (4.0–5.4)	484 (840151)
Male-bisexual	1.2 (0.9–1.6)	115 (216823)
Male-gay	0.9 (0.7–1.2)	83 (165310)
Male-not sure	4.7 (4.0–5.5)	456 (837259)
School type		
High school	45.3 (39.3–51.4)	4,018 (8338905)
Middle school	54.7 (48.6–60.7)	5,871 (10066635)
Race/Ethnicity		
Non-Hispanic White	47.5 (42.3–52.8)	4,260 (8522709)
Non-Hispanic Black	12.5 (10.0–15.4)	1,117 (2235054)
Hispanic	25.8 (21.8–30.1)	2,888 (4621087)
Other	14.2 (10.9–18.4)	1,340 (2554583)
Spoken language		
English	67.1 (62.6–71.3)	6,164 (12196524)
Other than English	32.9 (28.7–37.4)	3,606 (5983372)
Tobacco use of co-inhabitants		
No tobacco use	72.1 (69.8–74.4)	6,971 (12964667)
Combustible tobacco use	24.9 (22.9–27.1)	2,417 (4479665)
Race/Ethnicity and Tobacco use of co-habitants		
NHW & not living with tobacco user	32.8 (28.6–37.3)	2,828 (5754419)
NHB & not living with tobacco user	8.9 (7.1–11.2)	793 (1566524)
Hispanic & not living with tobacco user	19.9 (16.8–23.5)	2,152 (3496730)
Other & not living with tobacco user	10.4 (7.3–14.5)	1,014 (1820882)
NHW & living with combustible tobacco user	12.8 (11.2–14.5)	1,166 (2236630)
NHB & living with combustible tobacco user	3.2 (2.5–4.1)	277 (564472)
Hispanic & living with combustible tobacco user	5.4 (4.4–6.5)	624 (939106)
Other & living with combustible tobacco user	3.7 (3.0–4.5)	287 (647244)

NHW, Non-Hispanic White. NHB, Non-Hispanic Black.

### Prevalence of SHSe in homes

Overall, among students who do not use any form of tobacco, 84.9% (83.2–86.4) reported no SHSe, 7.5% (6.5–8.5) reported moderate SHSe, and 7.7% (6.8–8.6) reported severe SHSe in homes (Table 2). Non-Hispanic white students who lived with individuals who do not use any tobacco products had SHSe prevalence of 1.8% (1.3–2.5) and 0.5% (0.3–1.1) for moderate and severe SHSe in homes, respectively. On the other hand, these prevalences were 3.7% (2.2–6.2) and 2.7% (1.8–4.0) for non-Hispanic Black students and 3.1% (2.5–3.8) and 1.0% (0.6–1.7) for Hispanic students who lived with individuals who do not use any tobacco products, respectively. Among students who lived with combustible tobacco users, the moderate and severe SHSe prevalences in homes were 20.7% (17.5–24.2) and 29.0% (25.8–32.4) for non-Hispanic White individuals, 27.0% (19.0–36.8), and 31.4% (24.6–39.2) for non-Hispanic Black individuals, and 28.4% (22.8–34.8) and 21.8% (17.6–26.7) for Hispanics, respectively. For female students who self-identified as bisexuals, 11.6% (7.7–17.2) reported moderate, and 15.5% (11.2–21.0) reported severe SHSe. These SHSe prevalences are higher than those reported by heterosexual females, 6.2 (5.1–7.5) and 7.4% (6.4–8.5), respectively.

**Table 2:** Prevalence of moderate and severe SHSe in home and while riding in a vehicle by select characteristics of youth who have never used any form of tobacco—National Youth Tobacco Survey, 2020.

Characteristics	Moderate SSE in home	Severe SSE in home	*p*-value	Moderate SSE in vehicle	Severe SSE in vehicle	*p*-value
Overall	7.5 (6.5–8.5)	7.7 (6.8–8.6)		8.7 (7.7–9.7)	3.7 (3.1–4.4)	
Sex						
Female	6.7 (5.7–8.0)	8.4 (7.4–9.4)	0.005	8.7 (7.7–9.9)	3.9 (3.1–4.9)	0.733
Male	8.3 (7.1–9.6)	6.9 (6.0–8.0)		8.6 (7.3–10.2)	3.5 (2.9–4.3)	
Sexual identity						
Heterosexual	7.2 (6.3–8.3)	7.0 (6.2–8.0)	<0.001	8.2 (7.4–9.1)	3.4 (2.8–4.1)	0.001
Gay or lesbian	10.6 (6.3–17.3)	17.2 (11.9–24.3)		12.6 (7.0–21.6)	7.3 (4.0–12.7)	
Bisexual	11.0 (7.8–15.2)	14.1 (10.7–18.2)		11.8 (8.3–16.5)	7.0 (4.8–10.1)	
Not sure	6.8 (4.1–11.2)	6.8 (5.1–9.0)		9.7 (6.7–13.9)	3.3 (2.2–4.9)	
Sex-sexual identity						
Male-heterosexual	8.2 (7.1–9.6)	6.7 (5.7–8.0)	<0.001	7.8 (6.7–9.1)	3.3 (2.6–4.2)	0.007
Female-bisexual	11.6 (7.7–17.2)	15.5 (11.2–21.0)		10.6 (7.0–15.6)	6.7 (4.0–10.9)	
Female-gay or lesbian	10.7 (6.3–17.5)	18.7 (11.8–28.3)		10.2 (5.8–17.5)	5.5 (2.6–11.2)	
Female-heterosexual	6.2 (5.1–7.5)	7.4 (6.4–8.5)		8.7 (7.6–9.8)	3.6 (2.9–4.5)	
Female-not sure	6.1 (3.5–10.5)	7.1 (4.8–10.4)		8.6 (5.6–12.9)	3.6 (2.0–6.4)	
Male-bisexual	9.3 (5.1–16.2)	9.4 (4.6–18.3)		16.8 (9.8–27.4)	8.1 (3.0–20.3)	
Male-gay	11.0 (4.0–26.7)	14.0 (6.6–27.3)		16.3 (6.2–36.3)	10.0 (3.9–23.2)	
Male-not sure	7.5 (4.3–12.9)	6.4 (4.2–9.8)		10.9 (6.7–17.3)	3.0 (1.7–5.2)	
School type						
High School	6.8 (5.8–8.0)	7.9 (6.7–9.3)	0.359	8.6 (7.4–10.0)	3.8 (3.1–4.8)	0.887
Middle School	8.0 (6.7–9.7)	7.5 (6.4–8.8)		8.7 (7.5–10.1)	3.6 (2.9–4.5)	
Race/Ethnicity						
NHW	6.9 (5.7–8.2)	8.1 (6.8–9.6)	0.007	8.9 (7.7–10.1)	3.9 (3.0–5.1)	0.001
NHB	9.8 (6.9–13.9)	10.2 (8.3–12.4)		12.1 (9.6–15.1)	5.3 (4.1–6.9)	
Hispanic	8.4 (7.1–9.7)	5.5 (4.5–6.6)		7.5 (6.1–9.3)	3.0 (2.3–4.0)	
Other	5.7 (4.0–8.2)	8.3 (5.7–11.9)		6.7 (4.8–9.2)	2.4 (1.4–4.0)	
Spoken language						
English	7.4 (6.3–8.7)	8.5 (7.5–9.7)	0.006	9.2 (8.1–10.5)	4.1 (3.4–5.0)	0.009
Other than English	7.6 (6.2–9.2)	5.8 (4.9–6.9)		7.5 (6.1–9.1)	2.9 (2.2–3.7)	
Tobacco use of co-inhabitants						
No tobacco use	2.4 (2.0–2.9)	1.0 (0.7–1.4)	<0.001	4.2 (3.7–4.8)	0.8 (0.6–1.1)	<0.001
Combustible tobacco use	22.7 (20.3–25.3)	27.8 (25.8–29.9)		21.9 (19.5–24.6)	12.5 (10.7–14.5)	
Race/Ethnicity and Tobacco use of co-habitants						
NHW & not living with tobacco user	1.8 (1.3–2.5)	0.5 (0.3–1.1)	<0.001	3.6 (2.9–4.5)	0.6 (0.3–1.1)	<0.001
NHB & not living with tobacco user	3.7 (2.2–6.2)	2.7 (1.8–4.0)		5.4 (3.7–7.7)	2.0 (1.1–3.6)	
Hispanic & not living with tobacco user	3.1 (2.5–3.8)	1.0 (0.6–1.7)		4.4 (3.6–5.4)	0.7 (0.4–1.2)	
Other & not living with tobacco user	1.4 (0.7–2.7)	1.1 (0.5–2.4)		3.3 (2.4–4.5)	0.3 (0.1–1.3)	
NHW & living with combustible tobacco user	20.7 (17.5–24.2)	29.0 (25.8–32.4)		23.1 (20.4–26.1)	13.4 (10.8–16.5)	
NHB & living with combustible tobacco user	27.0 (19.0–36.8)	31.4 (24.6–39.2)		29.7 (23.1–37.2)	15.1 (11.0–20.3)	
Hispanic & living with combustible tobacco user	28.4 (22.8–34.8)	21.8 (17.6–26.7)		19.0 (13.7–25.6)	10.8 (7.7–15.1)	
Other & living with combustible tobacco user	18.5 (13.7–24.5)	29.0 (21.8–37.6)		16.7 (11.7–23.2)	8.6 (5.4–13.4)	

NHW, Non-Hispanic White. NHB, Non-Hispanic Black. *p*-value: Computed using the first and second-order Rao-Scott corrections to the Pearson chisquared test for survey data.

### Prevalence of SHSe while riding in a vehicle

The prevalence of SHSe while riding in a vehicle was 8.7% (7.7–9.7) and 3.7% (3.1–4.4) for moderate and severe SHSe, respectively (Table 2). Among students who lived with combustible tobacco users, the moderate and severe SHSe prevalences while riding in vehicles were 23.1% (20.4–26.1) and 13.4% (10.8–16.5) for non-Hispanic White individuals, 29.7% (23.1–37.2) and 15.1% (11.0–20.3) for non-Hispanic Black individuals, and 19.0% (13.7–25.6) and 10.8% (7.7–15.1) for Hispanics, respectively. Male students who identified themselves as gay reported 16.3% (6.2–36.3) moderate and 10.0% (3.9–23.2) severe SHSe, respectively, which was higher than moderate and severe SHSe reported by heterosexual males.

### Multinomial regression results for SHSe in homes

Results from the survey-weighted multinomial regression are reported in Table 3. Non-Hispanic Black students living with someone who does not use any form of tobacco products were significantly more likely to have moderate [OR = 2.1 (1.1–3.9), *p* = 0.03] and severe [OR = 5.1 (2.2–11.7), *p* < 0.001] SHSe in homes compared to non-Hispanic White individuals living with someone who does not use any form of tobacco products. Non-Hispanic White students living with individuals who use combustible tobacco products had 21.6-fold increased odds of moderate SHSe and 98.6-fold increased odds of severe SHSe in the home compared to non-Hispanic White students who lived with someone who did not use any tobacco products. Heterosexual female students had lower odds of reporting moderate SHSe in the home compared to heterosexual males [OR = 0.7 (0.6–0.99), *p* = 0.02], whereas bisexual females have two-fold odds of severe SHSe in homes compared to heterosexual males [OR = 2.0 (1.2–3.4), *p* = 0.01].

**Table 3:** Survey weighted multinomial regression of SHSe at homes and in vehicles among youth who have never used any form of tobacco—National Youth Tobacco Survey, 2020.

	SHSe in home				SHSe in vehicle			
	Moderate		Severe		Moderate		Severe	
	aOR	*p*-value	aOR	*p*-value	aOR	*p*-value	aOR	*p*-value
Age	0.9 (0.8–1.0)	0.14	0.9 (0.8–1.0)	0.09	1.0 (0.9–1.1)	0.89	1.0 (0.9–1.2)	0.61
Sex-sexual identity								
Male-heterosexual	Ref							
Female-bisexual	1.2 (0.7–2.1)	0.59	2.0 (1.2–3.4)	**0.01**	1.0 (0.6–1.7)	0.88	1.4 (0.7–2.7)	0.28
Female-gay or lesbian	1.1 (0.4–3.0)	0.78	2.4 (0.9–6.2)	0.08	1.0 (0.5–2.2)	0.99	1.2 (0.5–3.0)	0.63
Female-heterosexual	0.7 (0.6–0.99)	**0.02**	1.1 (0.9–1.4)	0.38	1.2 (1.0–1.5)	0.10	1.3 (0.9–1.8)	0.14
Female-not sure	0.6 (0.3–1.4)	0.24	1.0 (0.5–1.8)	0.97	1.2 (0.7–2.2)	0.43	1.4 (0.6–3.3)	0.40
Male-bisexual	0.7 (0.3–1.6)	0.40	0.8 (0.3–2.1)	0.67	1.7 (0.8–3.7)	0.18	2.2 (0.6–7.9)	0.21
Male-gay	1.3 (0.5–3.7)	0.56	1.8 (0.5–7.3)	0.38	2.4 (0.7–7.7)	0.14	2.7 (0.7–10.7)	0.15
Male-not sure	0.9 (0.5–1.9)	0.81	0.9 (0.5–1.8)	0.84	1.6 (0.9–3.0)	0.12	1.1 (0.5–2.5)	0.71
School type								
High School	Ref							
Middle School	1.0 (0.6–1.5)	0.87	0.7 (0.5–1.0)	0.07	1.0 (0.7–1.4)	0.83	1.1 (0.6–1.9)	0.77
Spoken language								
English	Ref							
Other than English	1.2 (0.9–1.6)	0.15	1.1 (0.9–1.4)	0.46	1.0 (0.8–1.2)	0.70	1.0 (0.7–1.4)	0.97
Race/Ethnicity and Tobacco use of co-inhabitants								
NHW & not living with tobacco user	Ref							
NHB & not living with tobacco user	2.1 (1.1–3.9)	**0.03**	5.1 (2.2–11.7)	**<0.001**	1.6 (1.0–2.6)	0.07	3.5 (1.5–8.0)	**0.005**
Hispanic & not living with tobacco user	1.5 (1.0–2.4)	0.06	1.7 (0.8–4.0)	0.17	1.3 (0.9–1.8)	0.16	1.1 (0.5–2.7)	0.75
Other & not living with tobacco user	0.7 (0.3–1.5)	0.33	1.9 (0.6–5.9)	0.23	0.9 (0.6–1.5)	0.71	0.5 (0.1–4.8)	0.55
NHW & living with combustible tobacco user	21.6 (14.8–31.6)	**<0.001**	98.6 (47.8–203.6)	**<0.001**	9.6 (7.3–12.6)	**<0.001**	33.2 (17.7–62.3)	**<0.001**
NHB & living with combustible tobacco user	36.3 (19.6–67.1)	**<0.001**	134.6 (63.8–284.0)	**<0.001**	14.7 (8.9–24.4)	**<0.001**	44.3 (19.3–101.6)	**<0.001**
Hispanic & living with combustible tobacco user	26.0 (16.8–40.5)	**<0.001**	68.7 (30.9–152.6)	**<0.001**	7.0 (4.4–11.2)	**<0.001**	22.5 (11.4–44.4)	**<0.001**
Other & living with combustible tobacco user	17.8 (10.9–29.0)	**<0.001**	93.5 (41.5–210.7)	**<0.001**	5.9 (3.7–9.5)	**<0.001**	18.3 (8.7–38.5)	**<0.001**

NHW, Non-Hispanic White. NHB, Non-Hispanic Black. Bold face indicates statistical significance at two-sided *p*-value ≤ 0.05. aOR, Adjusted Odds Ratio.

### Multinomial regression results for SHSe while riding in a vehicle

Non-Hispanic Black students living with someone who does not use any form of tobacco products were significantly more likely to report severe SHSe while riding in a vehicle compared to non-Hispanic White students living with someone who does not use any form of tobacco products [OR = 3.5 (1.5–8.0), *p* = 0.005] (Table 3). Non-Hispanic White students living with individuals who use combustible tobacco products had 9.6-fold increased odds of reporting moderate SHSe while riding in a vehicle and 33.2-fold increased odds of reporting severe SHSe compared to non-Hispanic White students who lived with someone who did not use any tobacco products.

## Discussion

This study reports the prevalence of youth SHSe in homes and while riding vehicles. Importantly, we identified disparities in SHSe among youth belonging to racial, sexual, and gender minority groups. Of concern, over 15% of the youth are exposed to SHSe in homes, and over 12% are exposed to SHSe in vehicles. Of the continuing concerns, among middle- and high-school youth living at homes where at least one member uses combustible tobacco, over 22% experience moderate and over 27% experience severe SHSe in homes. Similarly, while riding in a vehicle, approximately 22% experience moderate SHSe, and over 12% experience severe SHSe. Furthermore, non-Hispanic Black individuals and Hispanics were disproportionately affected by both moderate and severe SHSe, even when living with individuals who do not use any form of tobacco products. The disproportionate risk observed among racial and ethnic minorities could be because of several factors, including but not limited to lower knowledge of the hazards of SHSe, living in multi-housing units, and the mode of transportation utilized.

Our study also identified disparities in SHSe among sexual and gender minority youth. Although heterosexual females had a lower likelihood of SHSe, bisexual females were much more likely to be exposed to severe SHSe. Previous research has shown that sexual minorities tend to use tobacco products at a higher prevalence and at an earlier age than their heterosexual peers (20, 34, 35). Some studies have reported tobacco use patterns in gender minority youth, which suggests that younger cohorts of gender minority individuals may be particularly vulnerable (36, 37). We believe our findings of higher SHSe rates (e.g., bisexual female SHSe 27.1% compared to heterosexual female 13.6%) among non-smoking gender minority youth might be due to the social clustering of gender minority youth for social and emotional support (38). Furthermore, the stress associated with social stigma, discrimination, and targeted marketing by tobacco companies (39) has led to higher smoking prevalence in this community, leading to higher SHSe among non-smoking sexual minorities than their heterosexual counterparts.

Smoke-free laws prohibit smoking in public places; however, they do not include smoking bans in private vehicles and homes. Therefore, children have little choice but to continue being exposed to secondhand smoke. Several countries, including regions in Canada and the USA, have passed legislation banning smoking in private vehicles in the presence of children (40). A recent study assessing the impact of the smoking ban in cars with children in England and Scotland found that the ban led to a 72% relative reduction and a 4.1% absolute reduction in SHSe among children 13–15 years of age (41). Another study with 11–18 year olds reported a 22% relative reduction in children’s exposure to tobacco smoke in cars after accounting for the pre-existing declining trend (42). However, the stratified analyses revealed disparities in the impact of the policy, with significant reductions in exposure identified only among girls, younger children (aged 11–14), and those from less deprived backgrounds.

The observed disparities in the policy’s impact highlight the need for continued monitoring and evaluation of such interventions to identify and address any unequal effects on different populations. By recognizing and responding to these disparities, policymakers can work toward ensuring that all children, regardless of their race/ethnicity or other characteristics, benefit from the protection provided by bans on smoking in private vehicles.

The disparities observed in SHSe in the US may be due to significant gaps in home and car ownership by racial and ethnic minorities. In the second quarter of 2022, significant disparities in homeownership were evident: 75% of white households owned homes compared to 45 and 48% of Black and Hispanic households, respectively. These disparities worsen exposure to secondhand smoke, particularly among Black and Hispanic households who are more likely to live in rented multi-unit housing. Shared ventilation systems and spaces make it difficult to maintain smoke-free zones, exposing children and vulnerable residents to health risks, including SHSe, and compounding existing socioeconomic inequalities (43, 44). Similarly, minorities are less likely to have access to vehicles, with 18% of Black households lacking access compared to 6% of White households. This reduced mobility worsens secondhand smoke exposure disparities, as affected groups are more likely to rely on car-pooling and shared private vehicles, leading to a higher risk of SHSe (45).

Also, tobacco smoke leaves behind a persistent chemical residue, which consists of several toxic chemicals such as nicotine and polycyclic aromatic hydrocarbons. If smoked in homes and vehicles, these chemicals accumulate in significant concentrations on surfaces. This is referred to as thirdhand smoke (THS), and it interferes with the immune system and alters the normal microbiome of the individuals who get exposed (46, 47). Secondhand smoke (SHS) and thirdhand smoke (THS) differ significantly in their chemical makeup, physical properties, and exposure routes, making policies that effectively protect against SHS potentially ineffective against THS exposure (48). Policymakers should pay specific attention to THSe when enacting laws to reduce SHSe.

Public support for smoke-free housing is increasing, even among smokers, as the benefits of cleaner indoor air become more recognized. Future improvements could include extending stricter uniform smoke-free policies, including in private homes and vehicles across all states, stricter enforcement, and comprehensive educational campaigns. Public housing authorities could collaborate with health organizations to provide technical assistance and incentives for property owners, further strengthening smoke-free regulations and protecting residents from the dangers of SHSe (13).

By December 31, 2023, 28 states and several U.S. territories, including American Samoa, the District of Columbia, and Puerto Rico, have introduced regulations limiting smoking in worksites, childcare, and personal vehicles. However, despite growing public support and the proven benefits of these laws, there is still inconsistency in their application across states. Furthermore, smoking laws prohibiting smoking while children are around vary significantly, with some states protecting children under eight, while others protecting children up to age 18 (16).

Based on the high rates of SHSe observed in our study, we call for national legislation, similar to seat-belt laws, that prohibits smoking in private vehicles and homes where children are present. Standardizing these measures across states presents a challenge, which can be met through comprehensive public education promoting smoke-free households, including personal vehicles. Local and state governments and community organizations can work together to ensure consistent protection against secondhand smoke exposure, focusing on safeguarding children. Interactive health campaigns should engage the public through social media and community events, raising awareness about the dangers of secondhand smoke. Dynamic health warnings, including graphic health warning labels on tobacco products, can effectively communicate the risks of SHSe to children, encouraging smokers to adopt smoke-free behaviors in homes and vehicles when children are present. Additionally, smart detection devices can be used to monitor cigarette smoke levels in real-time and alert when exposure is detected, allowing for immediate action to mitigate the risk. Also, installing high-efficiency air purification systems in homes can significantly reduce secondhand smoke particles, reducing exposure to children.

### Limitations

This study is subject to some limitations. The NYTS is a self-reported survey and is, therefore, subject to recall and nonresponse bias. Also, the exposure is not verified with nicotine biomarkers. However, the validity of self-reported tobacco product exposure has been high in other population-based studies (49, 50) and has also been shown to consistently correlate well with serum cotinine levels (51). Additionally, the NYTS data is representative of middle and high school students who attended private or public schools; however, the study sample does not include school dropouts, another potential high-risk group. Nevertheless, according to the US Census Bureau School Enrollment Data (52), approximately 94% of children aged 10 to 18 were enrolled in traditional schools in 2019. Furthermore, our study did not have access to data on certain potential confounders, such as family economic status, parental education, and child health conditions. The absence of such variables may limit the deeper understanding of factors associated with youth SHSe.

Future research should prioritize longitudinal studies to estimate the causal relationships between SHSe and long-term health consequences in vulnerable populations, such as sexual/gender minority youth and those living with tobacco product users, as this is a limitation of cross-sectional studies. Such longitudinal studies should incorporate objective measures of SHSe, like cotinine levels in saliva or urine, which are reliable biomarkers of nicotine exposure from secondhand smoke (53). Additionally, future research should focus on developing and evaluating targeted interventions to reduce SHSe in vulnerable populations, including educational programs, enhanced funding for smoking cessation, and policies promoting smoke-free private houses and vehicles. Assessing the impact of these interventions on reducing cotinine levels and improving health outcomes will be crucial for informing evidence-based public health strategies to protect vulnerable populations from the harmful effects of SHSe.

## Conclusion

The study identified significant SHSe disparities among racial, sexual, and gender minority youth. Significant efforts are needed to develop targeted interventions to reduce SHSe in homes and vehicles, particularly in these vulnerable populations.

## Data availability statement

Publicly available datasets were analyzed in this study. This data can be found at: https://www.cdc.gov/tobacco/data_statistics/surveys/nyts/index.htm.

## Ethics statement

The Office of Management and Budget (OMB), ICF’s Institutional Review Board (IRB), and CDC’s Institutional Review Board (IRB) approved the NYTS cycles used in this study. The studies were conducted in accordance with the local legislation and institutional requirements. Written informed consent for participation in this study was provided by the participants’ legal guardians/next of kin.

## Author contributions

RT: Conceptualization, Investigation, Methodology, Validation, Writing – original draft, Writing – review & editing, Data curation, Formal analysis, Software. SahS: Investigation, Methodology, Validation, Writing – original draft, Writing – review & editing. SurS: Investigation, Methodology, Validation, Writing – original draft, Writing – review & editing. SanS: Investigation, Methodology, Validation, Writing – original draft, Writing – review & editing, Conceptualization, Funding acquisition, Project administration, Resources, Supervision.
